# Mortality in the City of New Orleans

**Published:** 1852-06

**Authors:** 


*Mortality in the city of New Orleans.*—A glance at the above table shows at once, that the *Zymotics* take the lead in the great work of death—amounting, in the aggregate, to 2,572. Next, in the magnitude of its numbers, is the class ranged under the diseases of the "*Nervous System*," being 1,278—a fraction above one half the deaths by the Zymotics. The third class in numbers, comes under the *Respiratory System*, reaching 1043. Hence, it appears that the Zymotic class outnumbers both the Nervous and Respiratory Systems.

The table also shows a very small mortality from diseases of the "Urinative System," (16) which can partly be explained on the ground of favo able climatic influences on our population. Again, can we not account for this remarkable exemption from serious diseases of the *urinative* organs, by reason of the vicarious activity of the cutaneous surface, superinduced by a warm, often humid, and for the most part, genial climate? That there exists a compensating sympathy between the skin and the urinative organs, is a well attested fact; and the "million" know that in warm weather, when the cutaneous surface eliminates a large amount of perspirable matter, the urinative organs are, in the same ratio, relieved in part of their accustomed burden. Hence, the simplicity and curability of affections of the urinative system in the latitude of New Orleans.

Of the 7275 deaths for the year 1851, in this city and Lafayette, 1871 perished in the Charity Hospital—more than three fourths of whom should be deducted from our resident population.

Although no deaths are embraced under the "Tegumentive" System—yet, perhaps, on a strict analysis of the causes of death, a few cases might be found under this head. We are persuaded, both from our own and the experience of other physicians of this city, that obstinate and chronic diseases of the tegumentary system are of infrequent occurrence in our climate. We sometimes meet wtih an imported case—which may have resisted treatment elsewhere—but even the same remedies which had previously failed in other climes, will succeed when brought to bear upon the disease in our latitude. Let those thus afflicted avail themselves of this important hint, and remember that if, as the Latin Poet observes, a change of climate does not always affectt he mind—yet, in many instances, it will aid essentially in modifying the covering of the body.—*N. O. Med. Journa.*

